# Varying variation: the effects of within- versus across-feature differences on relational category learning

**DOI:** 10.3389/fpsyg.2015.00129

**Published:** 2015-02-09

**Authors:** Katherine A. Livins, Michael J. Spivey, Leonidas A. A. Doumas

**Affiliations:** ^1^Department of Cognitive Science, University of California, Merced, Merced, CAUSA; ^2^School of Philosophy, Psychology and Language Sciences, University of Edinburgh, EdinburghUK

**Keywords:** category-learning, relational reasoning, feature variation

## Abstract

Learning of feature-based categories is known to interact with feature-variation in a variety of ways, depending on the type of variation (e.g., Markman and Maddox, 2003). However, relational categories are distinct from feature-based categories in that they determine membership based on structural similarities. As a result, the way that they interact with feature variation is unclear. This paper explores both experimental and computational data and argues that, despite its reliance on structural factors, relational category-learning should still be affected by the type of feature variation present during the learning process. It specifically suggests that *within-feature* and *across-feature* variation should produce different learning trajectories due to a difference in representational cost. The paper then uses the DORA model (Doumas et al., 2008) to discuss how this account might function in a cognitive system before presenting an experiment aimed at testing this account. The experiment was a relational category-learning task and was run on human participants and then simulated in DORA. Both sets of results indicated that learning a relational category from a training set with a lower amount of variation is easier, but that learning from a training set with increased within-feature variation is significantly less challenging than learning from a set with increased across-feature variation. These results support the claim that, like feature-based category-learning, relational category-learning is sensitive to the type of feature variation in the training set.

## INTRODUCTION

If one hears the words “ocean,” “mountain,” “hill,” “lake,” “river,” and “valley,” it is likely that one will separate the words into two groups—instances of bodies of water, and instances of land-based structures (possibly without even being prompted). The tendency to group things based on shared qualities is the basis for categorization—a process that humans perform quickly and frequently (e.g., Maddox et al., 2004). Categorization not only gives humans the ability to identify instances of a given category, but to also make predictions about novel items given knowledge of their category membership (Anderson, 1990; Markman and Ross, 2003). For example, categorization allows us to realize that we should be able to swim before we set about exploring an ocean, but that swimming is likely an unimportant ability if we intend to visit a mountain.

Strictly speaking, categories are sets; while set members may differ in some way, they share some common property (Markman and Maddox, 2003). That said, similarity is not the only important aspect of category knowledge and data has suggested that thinking about categories often relies on qualitative judgments about the relevancy of object properties and the degree to which objects share some set of them (Hammer et al., 2008, 2009a). As a result, categorization research has highlighted the importance of those properties, or *features* (Gentner and Kurtz, 2005). Typically, it has been found that category-learning becomes more challenging when features show greater amounts of variation across exemplars. For instance, Yamauchi and Markman (2000) used an experiment involving a family resemblance structure to show that irrelevant feature variation (i.e., a greater number of possible feature manifestations) made category-learning much harder, if not impossible.

There are exceptions though. Markman and Maddox (2003) pointed out that Yamauchi and Markman (2000) only exploited the variation of “exception features.” Exception features are non-typical of a category, but may be consistent with the prototype of another (Yamauchi and Markman, 2000). Variation in these features across exemplars means that the categories may appear more similar, thereby reducing one’s ability to delineate the categories. Thus, Markman and Maddox (2003) not only replicated the increased difficulty associated with exception features, but also explored how non-diagnostic feature variation (i.e., features that are not relevant or prototypical of any category) can affect category-learning. They found that this type of variation actually made category-learning easier. As a result, understanding the type of feature variation that a category-learning task is using, and the source of that variation, is important for predicting how it will affect participant performance.

However, as Markman and Stilwell (2001) and Gentner and Kurtz (2005) have pointed out, not all categories are built the same. There is a fundamental difference between a category of “blue things” and one of “things that are above other things”—the former can be evaluated by looking at an object, while the later requires one to evaluate the role that a thing plays in relation to another thing. The previously mentioned work (along with the majority of the existing category-learning literature) focuses on the former, which are called feature-based categories. This paper will deal with the later, which are called *relational categories*.

Relational categories are built on relational representations, which can be thought of as functions that assign some truth-value to an ordered k-tuple (see Gentner, 1989), and generally specify an actor and a patient. For example, “the dog chases the cat” is a relational statement that binds “dog” to the actor role, “cat” to the patient role, and can be evaluated as true or false. Importantly though, these bindings are dynamic, and the roles are independent of their fillers (Doumas et al., 2008). So, chasing means something independent of dogs and cats, and can take any number of objects as its elements (people, octopuses, political campaigns, etc.).

Thinking about relational categories, and relations in general, involves more or less ignoring the features of the objects in the class (i.e., the types of features that are typically important for most feature-based categorization; e.g., Markman and Stilwell, 2001). For example, while “blue things” comprise a feature-based category because all blue things share approximately the same hue, “above” things are defined by how they stand in relation to “below” things—their shared features can be sparse (or even non-existent) and are unimportant to their class membership. Thus, people, grades, cars, and octopuses can all equally be above other things and therefore included in the category, despite sharing few featural similarities.

There are at least two subtypes of relational categories (Markman and Stilwell, 2001; Gentner, 2005): (i) categories that involve objects that fulfill the same relational role, and so are engaged in the same relation (e.g., “things that are above other things”), and (ii) relational schemas that are defined by some internal relational structure that takes arguments (e.g., “transactions” are a class of situations which specify at least two individuals, and a good or service that is being transferred). However, both subtypes are role-focused instead of feature-focused and therefore fundamentally differ from feature-based categories.

Thus, while there is a wealth of literature on the complex ways that features and their variation across a category affects learning (e.g., also see Hammer et al., 2008, 2009a,b), there is little on how feature variation might affect relational category-learning. This gap in the literature is problematic because these two types of category-learning do not rely on equivalent mechanisms: relation-based category-learning relies on the difficult process of noticing and maintaining role-bindings (i.e., noticing which relational role an object fills, while potentially ignoring its features), while feature-based category-learning does not (Gentner and Kurtz, 2005; Doumas and Hummel, 2013). This gap is even more problematic with the realization that the literature is conflicted on the role that features play in relational reasoning in general.

**FIGURE 1:**
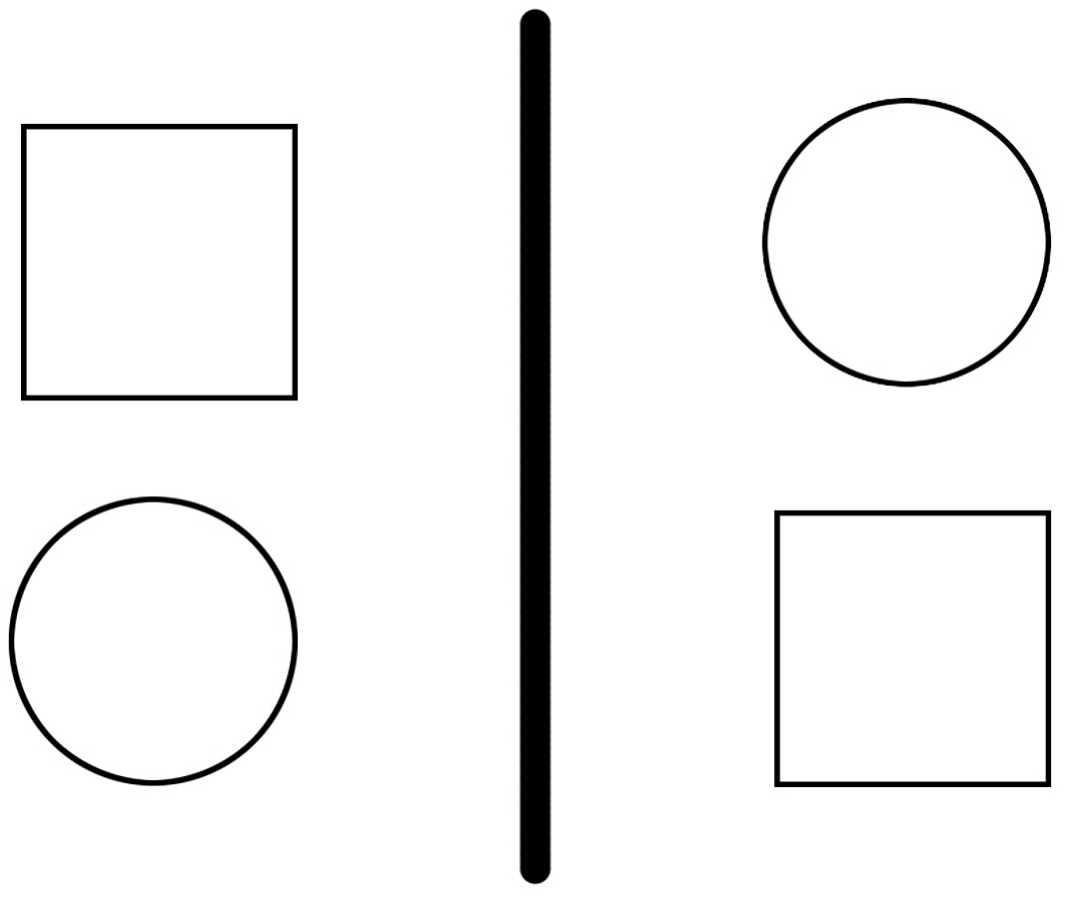
**An example of a cross-mapping problem.** Imagine that one is asked to map the shapes in the left pane to those in the right. A featural mapping would align the two squares and the two circles, while a relational mapping would align the top circle with the top square, and the bottom square with the bottom circle.

To the point, because relational reasoning relies on shared relational structures, object features can be distracting. For instance, cross-mappings are analogy problems that afford an answer (i.e., a mapping) based either on features or roles, but where the two result in different mappings (Gentner and Markman, 1997). So, if one is asked to complete the analogy task in **Figure 1**, and to map the two shapes on the left to the two shapes on the right, one could proceed in two different ways: (i) one could map featurally, by aligning the two squares and the two circles, or (ii) one could map relationally, by aligning the two “above” things [which can be described as mapping *above*(circle, square) to *above*(square, circle)]. While there is evidence to suggest that adult humans prefer relational mappings (Ratterman and Gentner, 1987; Markman and Gentner, 1993), distractions can promote featural mappings instead. For instance, Waltz et al. (2000) used time pressure to increase anxiety in their participants while they performed relational reasoning tasks that afforded either relational matches or feature matches; it was found that such conditions produced a greater number of feature mappings than relational mappings (also see Waltz et al., 1999 ^1^).

However, this is not to say that relations are devoid of features, nor that relations can be represented without them. In fact, it has been argued that features are important for learning relations in the first place. DORA, a computational model of relational development and reasoning (Doumas et al., 2008), suggests that relations are born out of experiences with objects, their features, and comparison. According to the model, feature variation can be detrimental during the initial learning of a relation, but some amount of variation is necessary to learn increasingly useful representations (Doumas et al., 2008). For instance, a child might learn the concept *big* by comparing a number of “big” things (e.g., a big bear and a big horse). Any extraneous features that vary systematically with the compared items will help the child make initial comparisons; however, those same features will also be part of the child’s *big* concept. So, *big* learned by comparing a bear and horse might also contain features like “animal,” “furry,” etc. Over time, the comparison of this *big* representation to other representations of *big* things with other sets of features, learned in other contexts (e.g., while comparing a big house and a big boat) will refine the child’s representation of *big* such that it begins to exclude extraneous features (such as “animal” and “furry”). Thus, according DORA, feature variation plays a fundamental role in the genesis of relational reasoning.

Furthermore, data from language learning suggests feature variation might be helpful for learning syntax. For example, Onnis et al. (2008) looked at the effects of variation sets in the acquisition of miniature artificial languages, and found that learning was improved by increasing the variation of the kinds of contexts a given lexical relationship showed up in. For example, if words C and D are frequently presented together in a training sequence, then the learner may eventually conclude that they form a structural phrase (i.e., simple bigram learning). However, if words C and D are frequently presented together, and in a widely varying set of contexts (e.g., BCD, CDE, ABCDE, CDEF, etc.), then the learner can quite quickly infer that CD forms a structural phrase. Thus, increasing the variation around a relationship can actually make it easier to learn that relationship. Although Onnis et al.’s (2008) task is not a traditional relational task, syntactic learning (like relational reasoning) is structure-sensitive. Furthermore, the task requires the extraction of structure from a feature-rich stream, without prior knowledge of which features belong to the syntax and which belong to the words instantiating the syntax—in other words, it requires one to reason about structure while ignoring many of the words and features.

In sum, while there exists a wealth of data on how feature-based categories interact with feature variation, feature-based categories differ from relational categories at a mechanistic level. However, it is unclear how the mechanisms involved in relational cognition interact with feature-variation, and so it is currently unknown how relational-category-learning might do so. Thus, while there is little dedicated research on the topic of feature variation in relational category-learning, there is good reason to believe that it is a factor that affects learning, and should therefore should be accounted for.

We propose that relational category-learning will be shaped by the *type* of feature variation exhibited across the training exemplars. To the point, there seems to be at least two different ways that exemplars might exhibit feature variation: first, they could demonstrate *within-feature* variation such that a single feature is represented in many different ways across exemplars. In this case low variation would involve fewer possible values of that feature across the training set, while greater variation would involve the opposite. Alternatively, they could demonstrate *across-feature* variation, such that more features could vary overall across exemplars. In this case, low variation would involve fewer varying properties.

**FIGURE 2:**
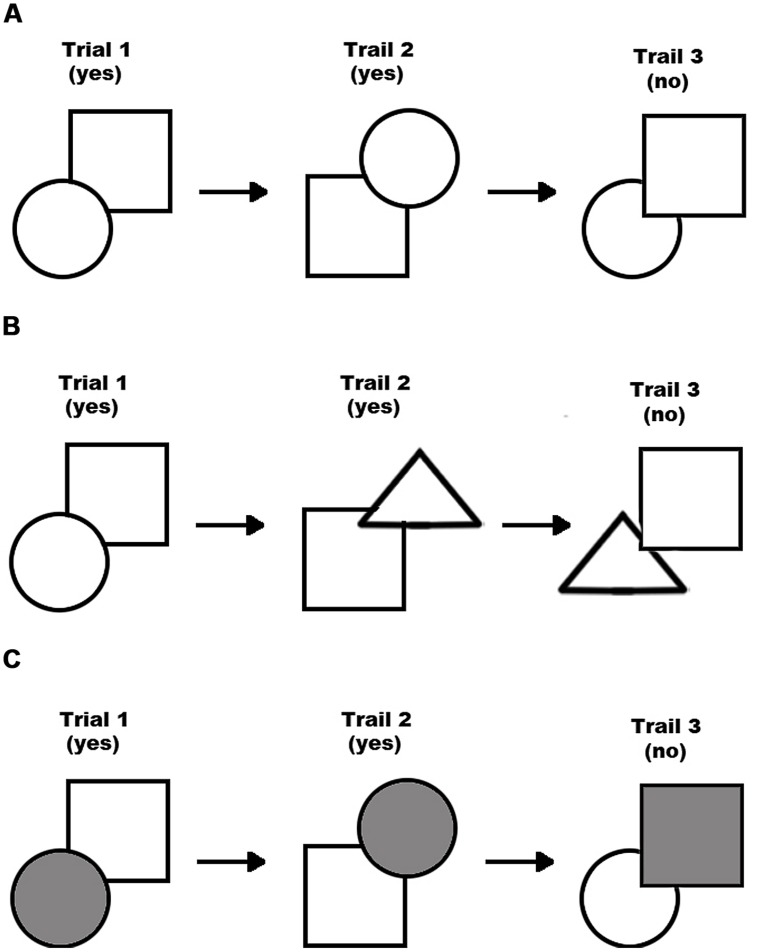
**An example of a hypothetical training set involving a base variation set (A), a within-feature variation set (B), and an across-feature variation set (C)**.

For instance, imagine that one needs to learn a relational category that involves the relative spatial locations of shapes such that membership is defined by whether one shape occludes the other at the top right of the occluded shape. Further imagine that while these shapes can be circles or squares, the actual shapes (i.e., which shape is in which location) is non-predictive of category membership – only the relational location structure is of importance (see **Figure 2A** for an example of what a training set for this category might look like). In this case, shape is a varying feature that is unimportant to category membership. Increased *within-feature* variation might mean increasing the number of shapes that could be used in the exemplars (so, instead of just circles and squares, the shapes might be circles, squares, or triangles; see **Figure 2B**). Alternatively, increased *across-feature* variation might be achieved by increasing the overall number of features that varied across exemplars. Thus, the shapes might vary, but now their colors might vary also (see **Figure 2C**).

We expect that learning relational categories involving higher levels of across-feature variation will have higher representational costs, and therefore be more difficult. Our logic is that when one begins learning a relational category without direction (beyond that a category must be learned), one has no choice but to begin by guessing. That guess must involve explicitly entertaining (i.e., representing) some feature or relationship exhibited by the stimulus, and testing it over multiple exemplars (given that people typically default to feature-based reasoning, this starting point will likely be featural; see Gentner, 1989; Ratterman and Gentner, 1998; Waltz et al., 2000; Doumas et al., 2008). If feedback is positive, then the category can be learned; if feedback is negative, then one can rule out that feature or relation as the category of interest and begin the process again with a different feature or relation.

While there is theoretically an infinite number of hypotheses that could be entertained, we expect that people will focus on properties or relations that vary between exemplars: after all, if they know that categories are generally defined by some set of shared properties, then between-exemplar differences will be information-baring and promote comparison. This expectation is supported by the feature-based category-learning literature, which suggests that determining relevant properties is a necessary step to learning (Rouder and Ratcliff, 2006; Hammer et al., 2008), and that comparison is generally an important part of that process (Hammer et al., 2008, 2009a,b). Furthermore, comparison seems especially important given that relational learning models like DORA predict that learning relies upon it (this point will be further described below; also see Doumas et al., 2008). So, we expect that people should notice a potential relevant property or relation, and then compare exemplars in order to determine whether it is category-defining. Thus, varying values within a feature would not add to the number of features that can be entertained as a possible category-marker, however, varying values across features means that one must potentially represent and rule out an extra possibility. This potential extra hypothesis makes it seem likely that the category would be harder to learn.

This paper will explore this account in three steps. First, it will use the DORA model to discuss how the extra representational cost potentially associated with across-domain variation might be represented in a cognitive system. DORA will not be presented as the only model that might be capable of this, however, it will be used because of its relational reasoning capabilities and, therefore, its ability to test how quickly categories can be learned while explicitly representing both relational roles per trial and hypotheses about category membership overall. It will then describe an experiment in which human participants are faced with different types of feature variation while performing a relational category-learning task. This task will look very much like the occluding-shape example used above, and will involve a feed-back based learning paradigm. The results will then be simulated in DORA so that model and human performance can be compared. Ultimately, it will be argued that the results from both the human and simulation data support the representational cost account.

### THE DORA MODEL AND OBJECT FEATURES

DORA is a symbolic-connectionist model that learns structured representations (like relations) from unstructured inputs (represented as feature vectors). DORA learns representations of predicates describing object properties and relational roles by comparing object feature vectors and learning explicit representations of any invariants that code for specific object properties or roles. By dynamically binding these representations of objects and predicates, DORA encodes features (e.g., information about an object’s properties) and structure (e.g., information about the role an object plays) at the same time, using the same representational material.

DORA codes for structured representations across layers of hierarchical nodes. In the bottom layer, a set of distributed feature nodes (called *semantic units*) encodes the features of objects and relational roles in a distributed fashion. At the next layer, localist *predicate/object* (PO) units combine sets of these features to represent particular objects and relational roles. Another layer of localist units called *role-binding* (RB) units then link those roles and objects, before an ultimate top layer of localist *proposition* (P) units link RB units into complete propositions (see **Figure 1**). For example, features such as “*y*-axis,” “high” and “object,” may combined at the PO level to represented *above-thing*, while “*y*-axis,” “low” and “object” may combine to create *below*. ^2^ At the next level of the hierarchy, roles and their arguments are then conjunctively coded at via RB units [i.e., *above*(square) + *below*(circle)], which are then ultimately combined via a *P* unit (at the top of the hierarchy of layers) to reify complete relational expressions such as “the square is above the circle” [or, *above*(square, circle)].

**FIGURE 3:**
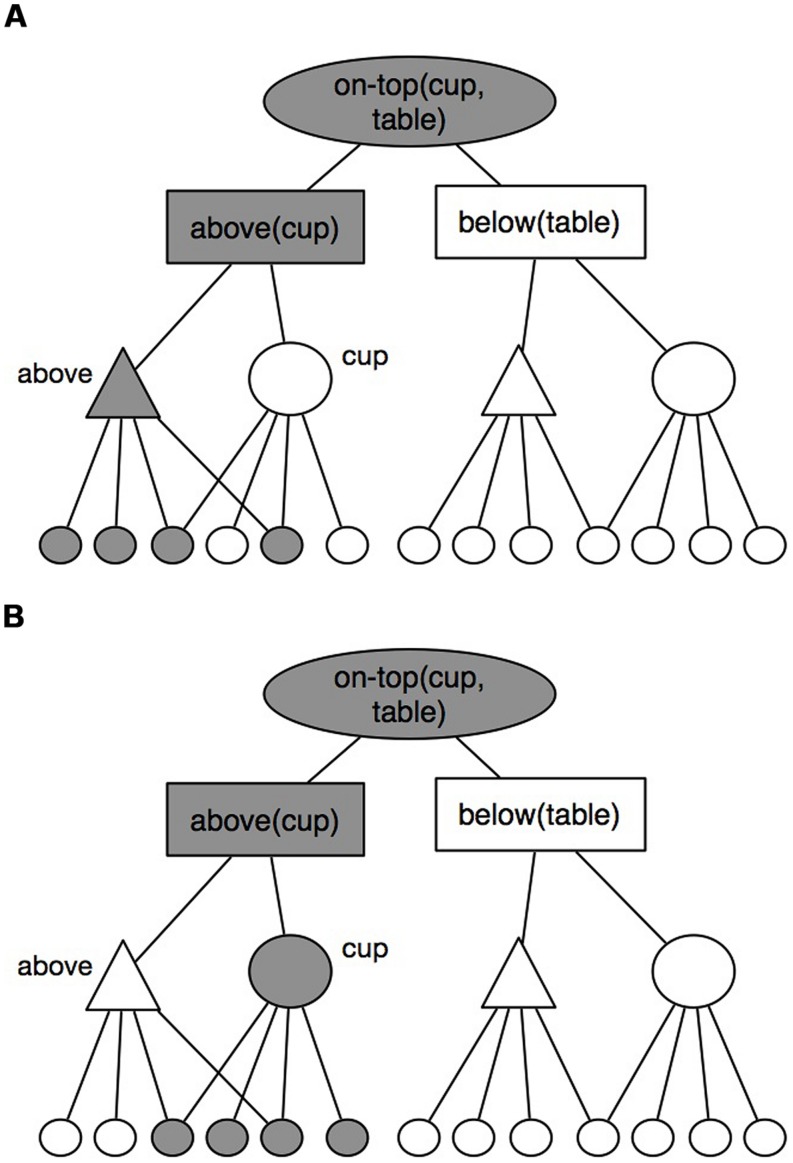
**An example of how DORA represents relational representations, and how those representations are fired across the model**. **(A)** shows the model firing the “above” role, while **(B)** shows it firing the “cup” filler.

While conjunctively coding bindings between roles and their fillers is sufficient for long-term storage, it fails catastrophically in accounting for relational processing (see, e.g., Doumas and Hummel, 2005). For the purposes of processing in working memory (WM), DORA, like the LISA model (Hummel and Holyoak, 1997, 2003) from which it is descended, uses time to carry binding information in WM processing. While LISA uses synchrony of role and filler firing to carry represent binding, DORA uses systematic asynchrony of firing (maintained at different levels of the hierarchy of layers representing relational propositions). For example, to represent the binding of square to *above* and circle to *below* while simultaneously keeping the semantics coding for role and their fillers distinct (a property necessary for learning new relations—see Doumas et al., 2008 for a more complete discussion), DORA might fire the nodes representing *above* followed directly by those representing square, and then the units represented *below* followed by those representing circle. Binding information is carried by proximity of firing (with bound roles and fillers firing in direct sequence), while the semantics of roles and fillers are not superimposed and are thus the representations of roles and their fillers remain independent (see **Figure 3**).

Unlike LISA, though, DORA accounts for the genesis of relational structures, while LISA begins at the point that those structures already exist. Specifically, DORA posits that comparison is central to the structure-learning process, allowing for shared properties or features to be highlighted and ultimately extracted (Doumas and Hummel, 2005; Doumas et al., 2008). In other words, it predicts that relations are learned by comparing many exemplars and extracting common features across them. During comparison, common properties become more active than properties that are exhibited by only a single example, which highlights invariants and starts a process by which DORA learns explicit predicate representations of these invariant properties (for a full discussion see Doumas et al., 2008).

For the current simulations, we made use DORA’s retrieval, mapping, and schema induction algorithms. These algorithms are very similar to the corresponding algorithms in LISA (differing only in that in DORA, they are specified for binding by asynchrony rather than synchrony). As a consequence, our reported simulations would almost certainly be possible in LISA as well.

#### Mapping

**FIGURE 4:**
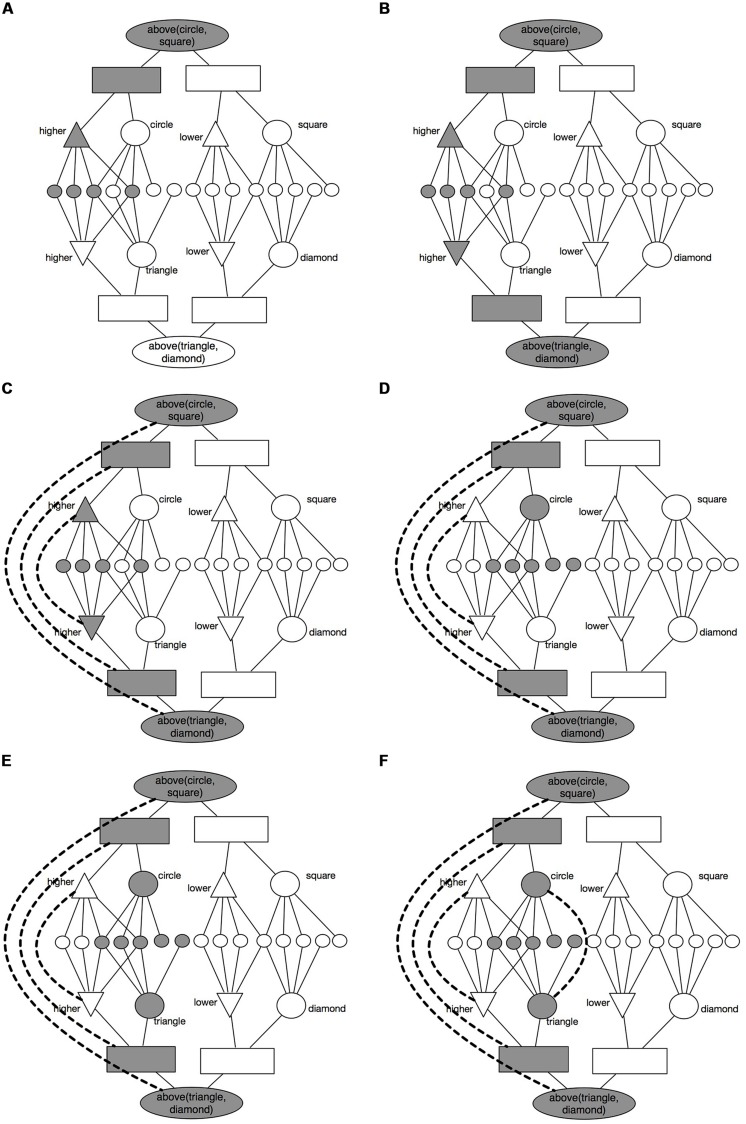
**An example of how DORA’s mapping algorithm works**.

DORA’s mapping algorithm is as follows. In DORA, items in WM are divided into two mutually exclusive sets for the purpose of making comparisons (see **Figure 4**). The *driver* is the current focus of DORA’s attention (i.e., what DORA is thinking about right now). The *recipient* is items in DORA’s active memory (e.g., Cowan, 2001) that are available for mapping to the driver. During processing, items in the driver become active and fire, and role-filler bindings are represented via systematic asynchrony (see above). So, if DORA is currently thinking about a circle above a square, the units representing *higher* might become active (see **Figure 4A**), followed by those representing circle (so specifying the binding of *higher* to circle; see **Figure 4D**), and then the units for *lower* fire followed by those representing square (so specifying the binding of *lower* to square). As units in the driver become active they impose a pattern of activation on the semantic units (e.g., when *higher* is active in the driver, the semantics of *higher* are active, and when circle is active in the driver, the semantics of circle are active). Units in the recipient complete via lateral inhibition to respond to the pattern of firing imposed on the semantic units by the active driver units. So, for instance, if *higher* is active in the driver, and a representation of *higher* is present in the recipient, because the two share so many semantics, the representation of *higher* in the recipient will tend to become highly active, thereby inhibiting the activation of other PO units in the recipient (see **Figures 4B,C**). DORA’s mapping algorithm is described fully in Doumas et al., 2008, but, in brief, when units of the same type (i.e., in the same corresponding layer) are active across driver and recipient, DORA attempts to map them establishing positive mapping weights between them in proportion to their mutual activation (see **Figure 4C**). Consequently, if two PO units across the driver and recipient are both highly co-active, DORA will form a strong mapping connection between them. The upshot is that DORA will tend to match similar items across driver and recipient based on shared features when no relations are present, and shared relations when such relations are present (Hummel and Holyoak, 1997 and Doumas et al., 2008 include full discussion of the algorithm and why it works).

#### Retrieval

The retrieval algorithm in DORA works very similarly, to mapping, except that when no items are in the recipient, items in long-term memory (LTM) are excited by the pattern of activation imposed on the semantic units by the units in the driver, and no mapping connections are formed. Rather, item in LTM become activated by the active semantic units, and are retrieved into the recipient via the Luce (1959) choice axiom.

#### Schema induction

After DORA has mapped items across the driver and recipient, it might attempt to learn a schema from the mapping. During schema induction, DORA fires the items in the driver, which in turn activate the items in the recipient to which they map. DORA then learns a representation of the overlap of the mapped PO units, that forms a more general version of the two mapped propositions. So, for example, if DORA has mapped *on-top* (cup, table) onto *on-tup*(hammer, toolbox), then as *supported* + cup and *supported* + hammar became active across the driver and recipient, DORA learns a new representation of their semantic overlap, or a representation roughly corresponding to *supported* + small-object. Similarly, when *supporter* + table and *supporter* + toolbox became active across the driver and recipient, DORA learns a new representation of their semantic overlap, or a representation roughly corresponding to *supporter* + big-object. (Again, full details of the schematisation algorithm and how it accounts for human schema induction data are given in Hummel and Holyoak, 2003 and Doumas et al., 2008).

That said, in DORA object properties do not *have* to be predicated—they can also be represented at the featural level of an object’s representation (i.e., in the semantic units). So, one might have a holistic representation of a big table, but not think about how “*big*” it is until one needs to fit it in a particularly small room. This ability for properties to be represented explicitly or implicitly (depending on the attention and goals of the system) is at the heart of how we predict feature-variation might affect performance during relational category-learning. Specifically, while an object might have a number of properties, those properties can be represented implicitly (i.e., at the semantic unit level).

In the following section we describe an experiment that we ultimately simulated in DORA. Both the human participants and DORA were given a relational category-learning task with different types of feature variation. We predicted that as features varied, they were more likely to be represented explicitly (i.e., as structured predicates) than features that that did not vary (which were more likely to be represented implicitly; i.e., as distributed features). We further expected that more across-feature variation (and therefore more explicit features) would result in slower learning times.

## EXPERIMENT

This experiment used a pictorial category-learning paradigm, which employed simple, spatial relations and geometric shapes. More specifically, participants were exposed to a number of exemplars, which depicted two shapes at a time, spatially aligned such that they could match a specific relational structure or not; the relational structure had to do with where one shape occluded another. Participants were not aware of what the relational structure was at the beginning of the experiment, so they would classify each exemplar, receive feedback, and ultimately learn the relational category through trial-and-error (see, e.g., Doumas and Hummel, 2013; Livins et al., under review).

This experiment specifically asks whether the type of feature variation has an effect on learning relational categories. Three conditions were used to this end: the first was a “base variation” condition, which varied shapes and their relative locations. The second was a “within-feature variation” condition, which increased the range of values that one of those features could take, while the third was an “across-feature variation” condition, which increased the number of features that varied overall.

Ultimately then, this experiment will help us to understand: (i) whether lower levels of variation do, in fact, help relational category-learning, and (ii) whether increased within-feature and across-feature variation affect relational category-learning in the same way.

### PARTICIPANTS

After attaining ethics approval from the University of California Merced’s IRB, participants were collected and included 137 undergraduate students from that university. They were recruited through the school’s participant pool and received course credit for their participation. All participants were required to have normal or corrected-to-normal vision.

The study was an “online” study, and was hosted on an independent website, such that participants could complete the study at any time or from any location. The online system functions analogously to Amazon’s Mechanical Turk, and was expected to produce the same type of data. Such data has been found to be equally reliable to traditional in-lab methods (see Buhrmester et al., 2011).

### DESIGN

Participants were randomly assigned to one of the three conditions. All conditions began by having their web browser directed to an independent website hosting the experiment. The first page of the site held the instructions, which told participants that they were going to be shown a series of pairs of shapes, and that the shapes were positioned according to some “rule.” It also stated that they were not going to be told the “rule,” but that they needed to determine it through the feedback provided along with trial-and-error.

Participants then began the “training phase,” during which they would learn a relational category. At the start of this phase, their browser was redirected to a new page, which presented a gray box in the center of the participant’s browser window on a white background. The box was 700 by 500 pixels in size. At the beginning of every trial, participants would see a fixation cross in the center of the box for 1500 ms, then an exemplar stimulus, which would be displayed in the center of the box. Participants would then respond by key press as to whether the exemplar followed the “rule” or not. In every case, they had as much time as needed to respond.

**FIGURE 5:**
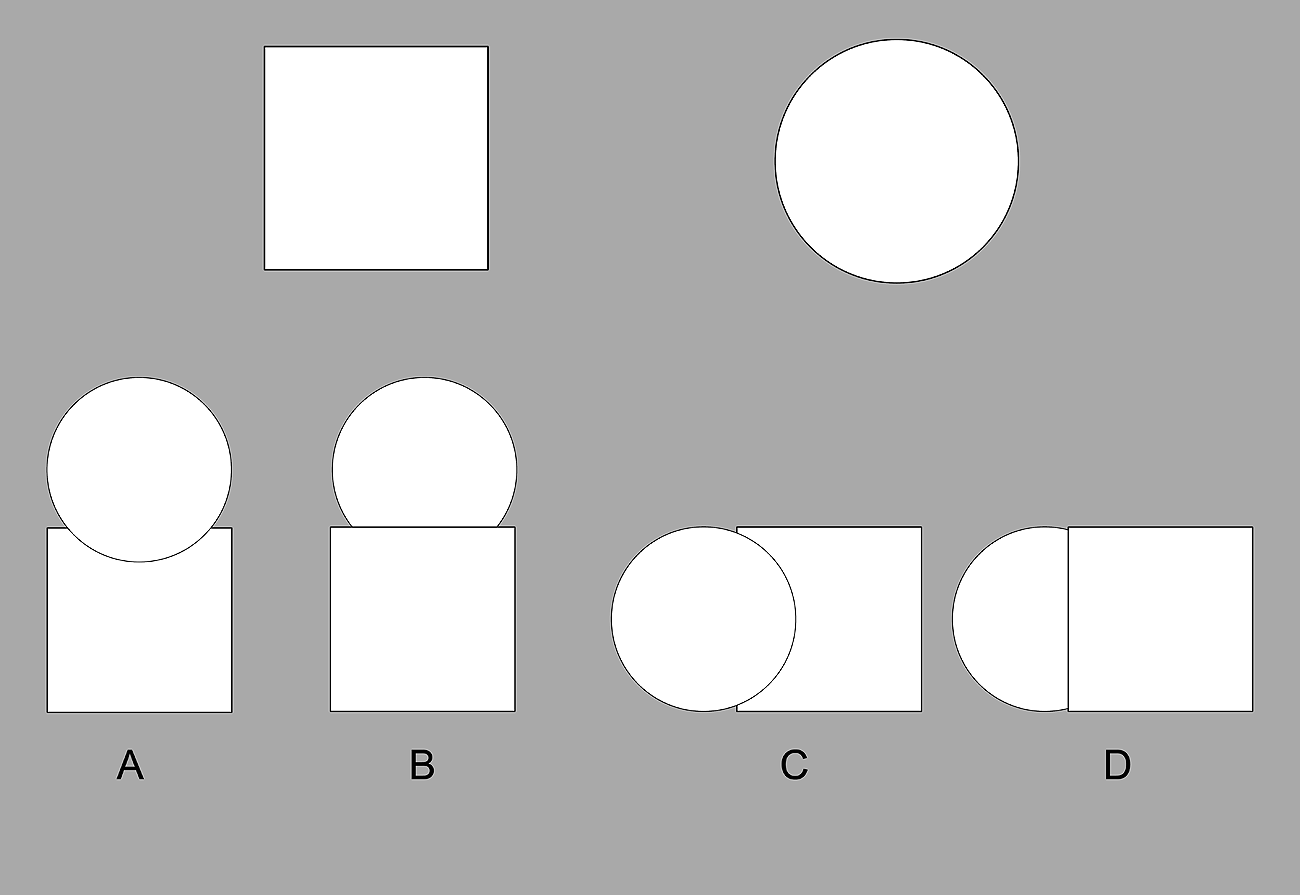
**An example of how two shapes may combine to create exemplars that have an occluder that takes a value on the “left/right” feature or the “above/below” feature.** The “left/right” feature allowed for an exemplar to be classified as an “A” or a “B” depending on the occluder’s placement, while the “above/below” feature allowed for an exemplar to be classified as a “C” or a “D” depending on the occluder’s placement.

The exemplars, and their relational categories, were created using simple shapes (e.g., circles and squares) and their relative placement on the *x* and *y*-axes. More specifically, every exemplar stimulus showed two shapes, where one shape partially occluded the other. Category membership was decided by the location of the occluding shape. Every occluder (in every condition) took a value on two different relations: it could be slightly to the left or right of the occluded shape, and it could be slightly above or below it. Thus, every exemplar could be thought of as an “A” if the occluder was above the occluded shape, a “B” if the occluder was below the occluded shape, a “C” if it was to the left of the occluded shape, and a “D” if was to the right of the occluded shape (see **Figure 5**).

**FIGURE 6:**
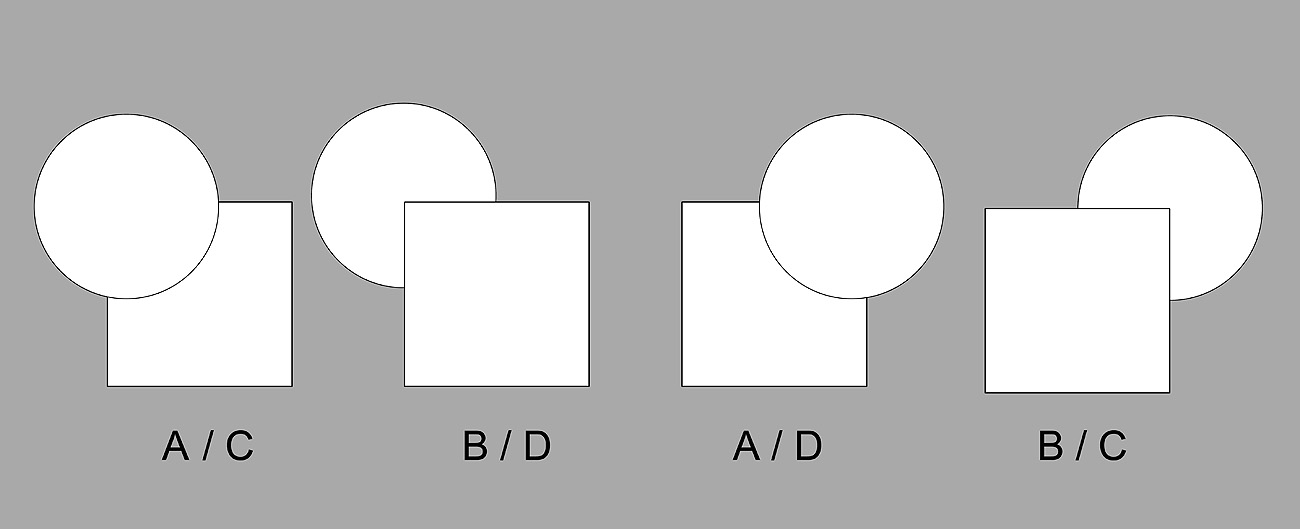
**Examples of exemplars that combine a value on the left/right relation with a value on the above/below relation.** As depicted, each exemplar takes a value on both relations. A/C has the occluder above and to the left of the occluded object, B/D has it below and to the right of it, A/D has it above and to the right, and B/C has it below and to the left.

Combining values on the two relations allowed for the creation of relationally ambiguous stimuli since every exemplar would simultaneously exhibit more than one relation. More specifically, A/C pairings depicted an occluder that was above and to the left of the occluded shape, B/D pairings depicted an occluder that was to the bottom and to the right of the occluded shape, A/D pairings that depicted an occluder that was to the top and to right of the occluded shape, and B/C pairings that depicted an occluder to the bottom and to the left of the occluded shape (see **Figure 6**).

Importantly though, relational categories were systematically paired during training such that half of the participants saw the A/C combinations and B/D combinations, while the other half of the participants saw the A/D combinations and B/C combinations. Thus, the training phase for each participant began by randomly selecting a *pair* of training rules that conflated a relative location on the horizontal axis with a relative location on the vertical axis (i.e., A/C and B/D, or A/D and B/C). One rule pair would be randomly associated with a left-key, and the other to a right-key, such that every exemplar stimulus could be classified dichotomously. Participants were told whether they were “correct” or “incorrect” after every key press.

Given that each exemplar conflated a value on two different relations, participants in all conditions could learn a horizontal rule, a vertical rule, or both rules. For instance, if a participant’s training rules were A/C and B/D, where A/C was assigned to the left-key, then she could learn one of three things: (i) that the left-key needed to be pressed whenever the occluder was to the left of the occluded shape, (ii) that left-key needed to be pressed whenever the occluder was above the occluded shape, or (iii) that left-key needed to be pressed whenever the occluder was above and to the right of the occluded shape.

The specific shapes in a given trial were selected at random, creating non-predictive shape selections; however, the specific shapes did vary by condition. To the point, the base-variation condition had circles and squares, each white with a 2-pixel black outline. Each trial randomly assigned a circle or a square to be the occluding shape, and a circle or a square to be the occluded shape. As a result, each trial could contain a circle and a square, a circle and a circle, or a square and a square. The within-feature variation condition increased the number of values that the “shape” feature could take. Thus, it had a greater number of shapes that could vary, including circles, squares, triangles, and hexagons. Like in the base-variation condition, each shape could appear with itself, or any other shape. The across-feature variation condition matched the base-variation condition on shape variation (i.e., it had circles and squares), but added color as a varying feature. The colors included white and dark purple (both of which had a black outline, like in the other conditions). The colors of each shape randomly varied like the shapes themselves, and so trials could include a white and a purple shape in either occluding role, or alternatively two shapes of the same color.

There are three important things to note about these conditions. First, the variation in each condition was achieved across trials. Thus, any single exemplar in all conditions necessarily had more features than noted above – for instance, shape size, opacity, etc. However, these features did not vary across exemplars or conditions, and so their presence or absence could not account for performance differences. Second, the two higher-variation conditions displayed an equal number of possible feature-value combinations: the within-feature variation condition allowed for each shape to be presented along with a copy of itself, and with each other shape, totaling 10 possible feature combinations. Likewise, the across-feature variation condition allowed each color to be represented in each shape, in each color, also totaling 10 possible combinations (see **Table 1** for possible combinations). Thus, performance differences could not be due to combinatorial differences and the extra variation that would come with such differences—the only difference between the conditions was the number of varying features, and the number or type of manifestations that those features could instantiate. Finally, in all cases, the shapes and their colors were non-predictive of the relational rule, and so were not predictive of category membership.

**Table 1:** A table depicting all possible shape combinations that a participant could see by condition.

Condition	Feature combinations
Base variation	Circle + Square | Circle + Circle | Square + Square
Within-feature variation	Circle + Circle | Square + Square | Triangle + Triangle | Pentagon + Pentagon | Circle + Square | Circle + Triangle | Circle + Pentagon | Square + Triangle | Square + Pentagon | Triangle + Pentagon
Across-feature variation	White Circle + White Circle | White Square + White Square | Purple Circle + Purple Circle | Purple Square + Purple Square | White Circle + White Square | Purple Circle + Purple Square | White Circle + Purple Square | Purple Circle + White Square | White Circle + Purple Circle | White Square + Purple Square

Participants continued to see pairs of shapes, and get feedback until they learned a rule well enough to correctly classify ten trials in a row. If a participant answered a trial incorrectly, the counter reset to zero. The counter was displayed at the top of the screen as a participant progressed. If the participant failed to reach criterion in 100 trials or less, they were directed to a finishing screen and completed no further classification tasks.

If the participant did reach criterion, then she moved on to the “testing phase.” She was told that she would continue to see pairs of shapes, but that all feedback as to whether she was correct or incorrect would stop. Participants were also instructed to continue to use the same rule that they had learned during training for the remainder of the experiment.

The test phase then began and participants were presented with a random order of 28 trials. These trials were made up of seven exemplars of each possible category combination, such that they included A/C, A/D, B/C, and B/D shape alignments. Thus, half of the test phase required participants to replicate their ability to classify the exemplars of their training rules, and the other half required them to generalize that rule to novel exemplars. Furthermore, participants’ responses on the novel exemplars also indicated which rule they learned, since it would indicate which training category the participant thought the novel pair was like, and therefore whether they learned the “above” relational category or the “beside” relational category.

**FIGURE 7:**
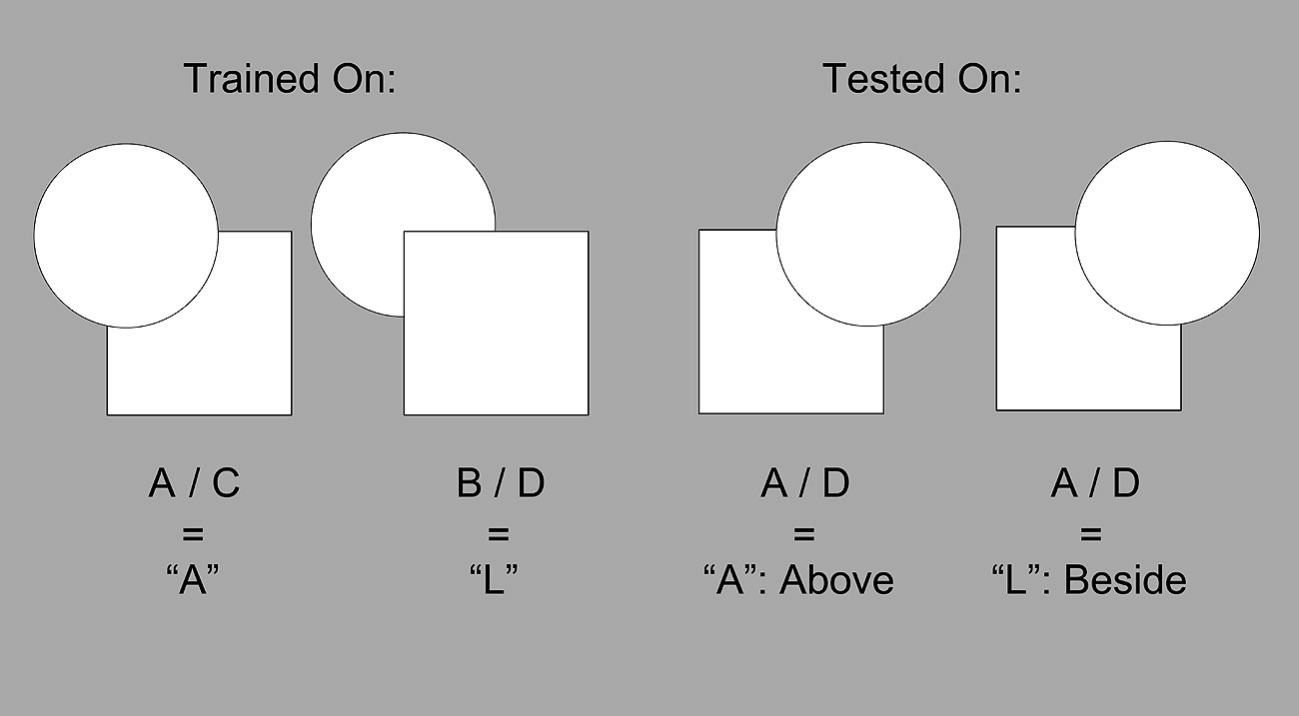
**An example of a possible training set and test phase exemplar.** Imagine the participant was trained on A/C and B/D exemplars, where an “*left-key*” press was paired with A/C, and an “*right-key*” press was paired with B/D. If a participant were then shown an A/D exemplar during the test phase, then an “*left-key*” press would indicate that A/D was classified in the same way as an A/C exemplar, while a “*right-key*” press would indicate that it was classified in the same way as a B/D.

For example, suppose that a participant had been trained on A/C and B/D, where A/C had been associated with a left-key press, and B/D had been associated with a right-key press. For the generalization portion of the testing phase, novel A/D and B/C pairs could be used to determine which rule the participant had learned: if presented with an A/D pairing, then a left-key press would indicate that the participant was classifying the stimulus to be like the A/C pair. If A/C and A/D pairs are classified in the same way, then the participant must be attending to the above/below relation (since the left-key is the common relational value between them, representing cases where the occluder was above the occluded shape). Along the same lines, a right-key press would indicate that the participant was classifying by the “beside” rule (See **Figure 7**). While the study was not primarily concerned with which rule participants learned, the ability to determine which rule each participant learned was instrumental in determining generalization accuracy and ability (i.e., if they learned one rule, how accurately can they classify the novel exemplars by that rule?).

Once testing was complete, participants were directed to a final screen where they were asked to type in the rule that they learned in fifty characters or less.

This design allowed us to collect data on four measures of relational category-learning. First, we measured whether participants learned any rule at all within 100 trials (i.e., overall rule learning). Second, for those that did learn a rule within 100 trials, we measured the number of trials required to learn that rule (i.e., speed of rule acquisition). Third, we measured the speed at which participants reacted during rule learning (i.e., response latency of classification while learning). And finally, we measured the degree to which participants could accurately generalize the rule to novel exemplars during the test phase (i.e., testing robustness).

### RESULTS

A participant’s performance on trained exemplars during the testing phase was used to determine whether she had learned the rule well enough to continue applying it without feedback. Thus, a participant was classified as a “rule learner” if she: (i) achieved the criterion of 10 consecutive correct responses during the training phase, and ii) did not incorrectly classify more than four of the 14 exemplars representing their training rules during the test trials (i.e., she accurately classified training exemplars with more than 71% accuracy). Any participant that did not meet these two criteria was classified as a “non-rule learner” for subsequent analysis.

The only exceptions to these criteria were the dual-rule learners (i.e., those that were considered to have learned both rules) since their test data can look analogous to participants that learned nothing at all. As a result, we relied on their typed answer of a rule. Thus, they were considered to have learned both rules only if they met the above criteria, and also explicitly stated that they learned both the above/below relation and the beside relation. With that criterion in mind, we can specify how participants performed on the four measures outlined in the experiment.

**FIGURE 8:**
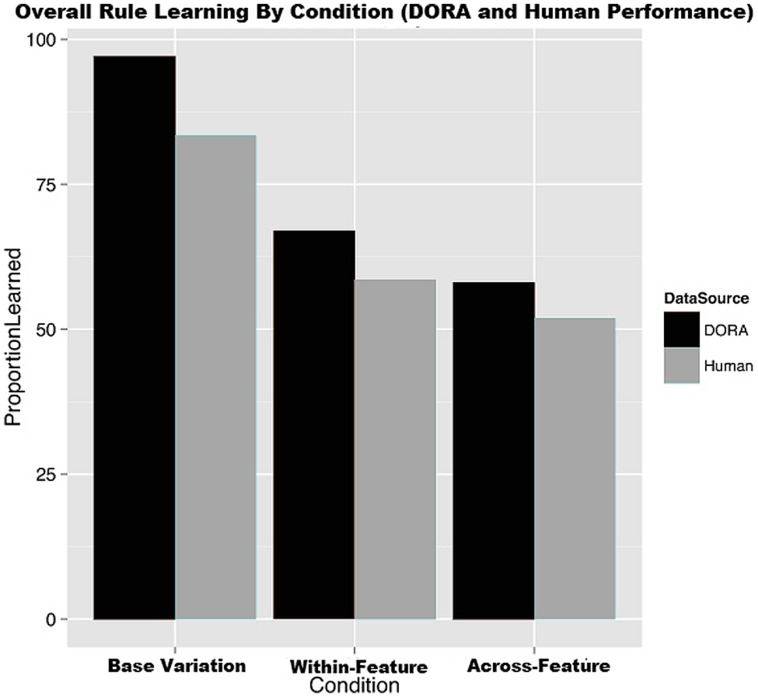
**The proportion of rule learners organized by condition for both DORA and the human participants**.

First, with regard to overall rule learning, a chi-square test revealed that there was a significant difference in how many participants learned a rule across conditions [χ(2) = 10.725, *p* < 0.01]. In detail, the base-variation condition had 7 of its 42 participants (16.67%) fail to learn, the within-feature variation condition had 17 out of its 41 participants (41.46%) fail to learn, and the across-feature variation condition had 26 of its 54 participants (48.14%) fail to learn (see the “human data” portion of **Figure 8**). These values suggest that learning is much easier in the base-variation condition than in the two other conditions that increase variation in the stimuli.

At this point, it is important to notice that further calculations involving the data from non-rule learners are impossible: each of the following statistics will involve comparing performance during learning in order to determine which condition produced the greatest levels of learning difficulty. However, non-rule learners never actually learned anything, and so such values are impossible to predict. As a result, non-rule learning participants have been removed from further statistical analyses.

**FIGURE 9:**
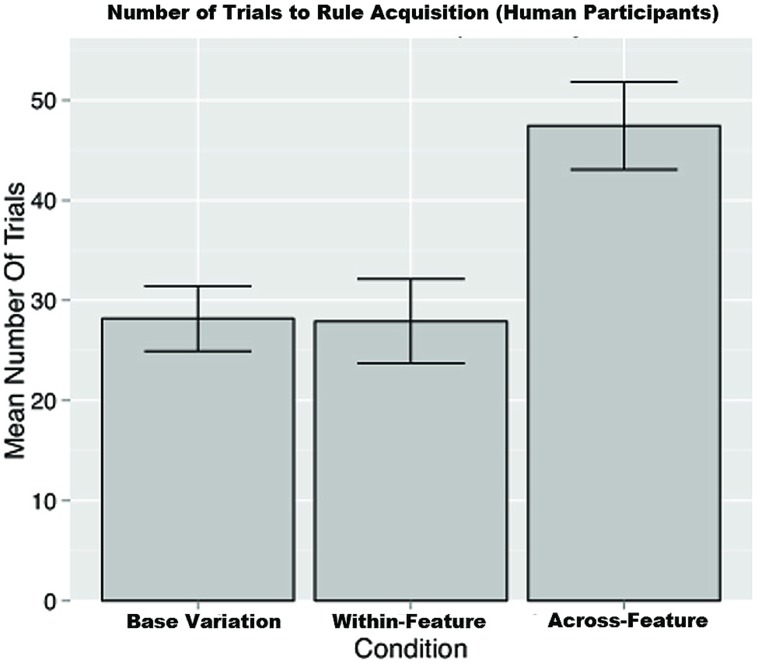
**The mean number of trials required for rule acquisition by condition in the human conditions.** Error bars represent the means plus or minus the SEs.

Second, with regard to the number of trials required for rule acquisition, a one-way ANOVA revealed that the number of trials that participants took to learn the rule was significantly different across conditions [*F*(2,84) = 7.420, *p* < 0.01]. A Bonferroni *post hoc* test showed that this difference was non-significant (*p* = 1.00) between the base-variation condition (*M =* 29.09, SD = 20.32) and the within- feature variation condition (*M* = 27.92, SD = 20.60), however, the base-variation and the within- feature variation conditions both showed significantly faster acquisition rates (*p <* 0.01 in both cases) than the across- feature variation condition (*M* = 47.43, SD = 23.08; see **Figure 9**).

**FIGURE 10:**
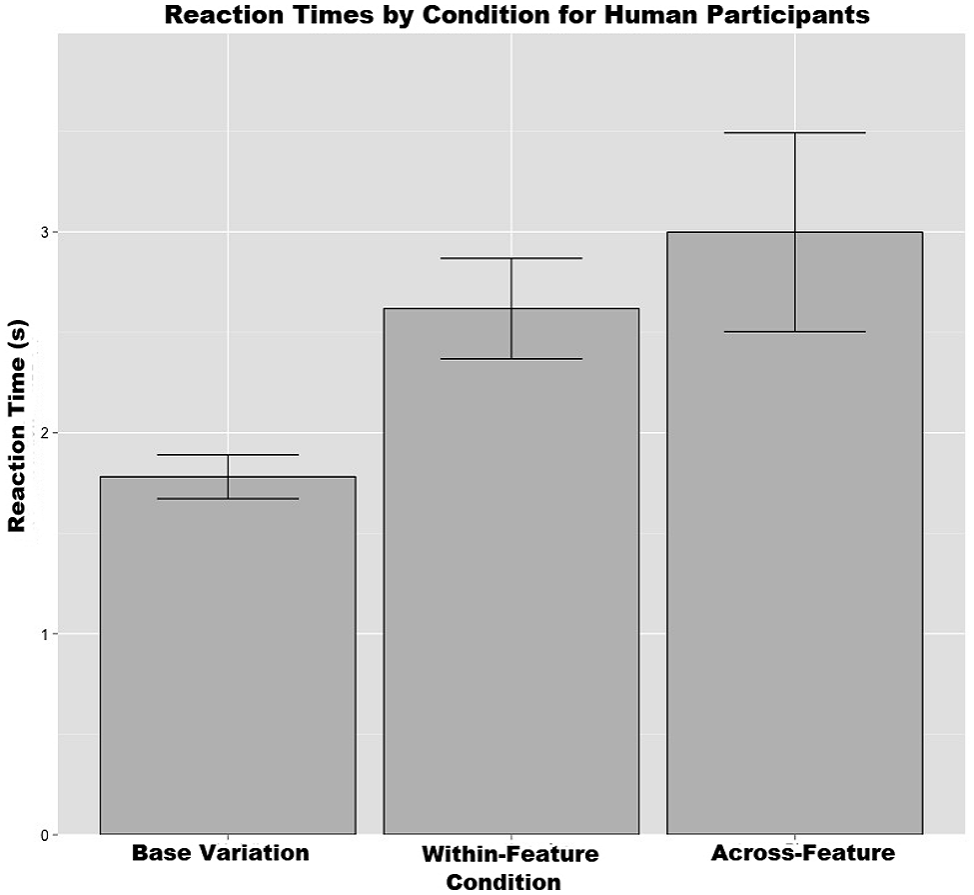
**The mean reaction times during training trails organized by condition.** Error bars represent the means plus or minus the SEs.

Third, with regard to reaction times during the training trails, after reaction times more than 3 SDs from the mean were removed, another one-way ANOVA showed that there was a significant difference between conditions [*F*(2,84) = 4.414, *p* < 0.05]. A Bonferroni *post hoc* test showed that the base-variation condition (*M* = 1.78, SD = 0.65) had a significantly faster mean reaction time than the across-feature variation condition (*M* = 3.00, SD = 2.61; *p* < 0.05), however, it was not significantly faster than the within-feature variation condition (*M* = 2.62, SD = 1.22; *p* = 0.37). There was also no significant difference between the within-feature and across-feature conditions for this measure (*p* = 0.185; see **Figure 10**).

Finally, with regard to performance on the generalization questions during the testing phase (i.e., those with novel exemplars), it was found that accuracy rates by condition were not significantly different (*M* = 12.54, SD = 1.54 for the base-variation condition; *M* = 12.37, SD = 1.91 for the within-feature condition;*M* = 12.86, SD = 1.86 for the across-feature condition; a one-way ANOVA showed that *F*(2,84) = 0.518, *p* = 0.597.

## THE SIMULATIONS

We modeled the previously described category-learning paradigm in DORA. Again, we do not claim that DORA is the only model that may account for these results; rather, our claim is simply that varying an extraneous feature’s variability makes a relational category harder to learn, and that across-feature variation has a more negative impact on learning than within-feature variation.

The simulation proceeded as follows. DORA began with a repository of representations stored in LTM. These representations included predicates describing object properties (e.g., color and shape) and spatial relations [e.g., *next-to*(*x*, *y*) and *above* (*x*, *y*)] ^3^. On each trial we represented the current problem in the driver using a set of relevant predicates (see below) sampled from LTM. DORA then attempted to retrieve previous exemplars from LTM to match the current exemplar, and finally attempted to map the current exemplar on to those retrieved exemplars. If DORA managed to map the current exemplar onto a previously seen exemplar, it “answered” with the category of the mapped previous exemplar. If DORA was correct, it generated a schema based on the mapping (i.e., it tried to create a more general representation of a category member). If it was incorrect, it simply recorded the category label of the current exemplar as a semantic unit and stored the exemplar in LTM.

In the “base variation” condition, only shapes and their relative locations varied. To simulate this condition, each new exemplar could be coded using predicates describing its relative location and its shape, and an implicit feature (i.e., a semantic unit) describing its color. In the “within-feature variation” condition the range of values that the “shape” feature could take was increased. To simulate this condition, the current exemplar could be coded with predicates describing its spatial location, its shape (with the greater range), and an implicit property representing its color. Finally, in the “across-feature variation” condition the number of features that varied overall increased. To simulate this condition, the current exemplar could be coded using any of three explicit predicates (e.g., shapes, their relative locations, and colors). The structure of our simulations followed from our assumption that the predicating (i.e., explicitly representing) a particular feature is more likely when that feature has some variation. So, in a condition where all shapes were white, predicating color was more unlikely than in a condition were color varied between shapes. This assumption rests on empirical results indicating that explicitly predicating the value on a feature is much more likely when two items with different values on that feature are compared (see Doumas and Hummel, 2013). The assumption also follows from DORA’s processing algorithm, where detecting a difference requires that compared objects do not share some particular feature. For each of the three conditions, we ran 100 iterations of learning instances, simulating 100 participants per condition.

DORA went through a series of learning iterations (epochs) until the model succeeded on 10 classifications in a row (i.e., it mapped to the correct relational category) or until it reached 100 epochs. DORAs performance on the two measures that were significant in the human data were tracked— whether or not learning occurred (by meeting the classification criterion) and the number of trials taken to meeting criterion.

### RESULTS

Mirroring the human data, DORA performed best in the base-feature variation condition, moderately in the within-feature variation condition, and worst in the across-feature variation condition on both measures. Specifically, the base-variation condition took an average of 23 trials to reach criterion during training, with a 97% overall learning rate; the within-feature variation condition took an average of 38 trials to reach criterion, with a 67% overall learning rate; and finally, the across-feature variation condition took an average of 51 trials to reach criterion, with only a 58% overall learning rate.

**FIGURE 11:**
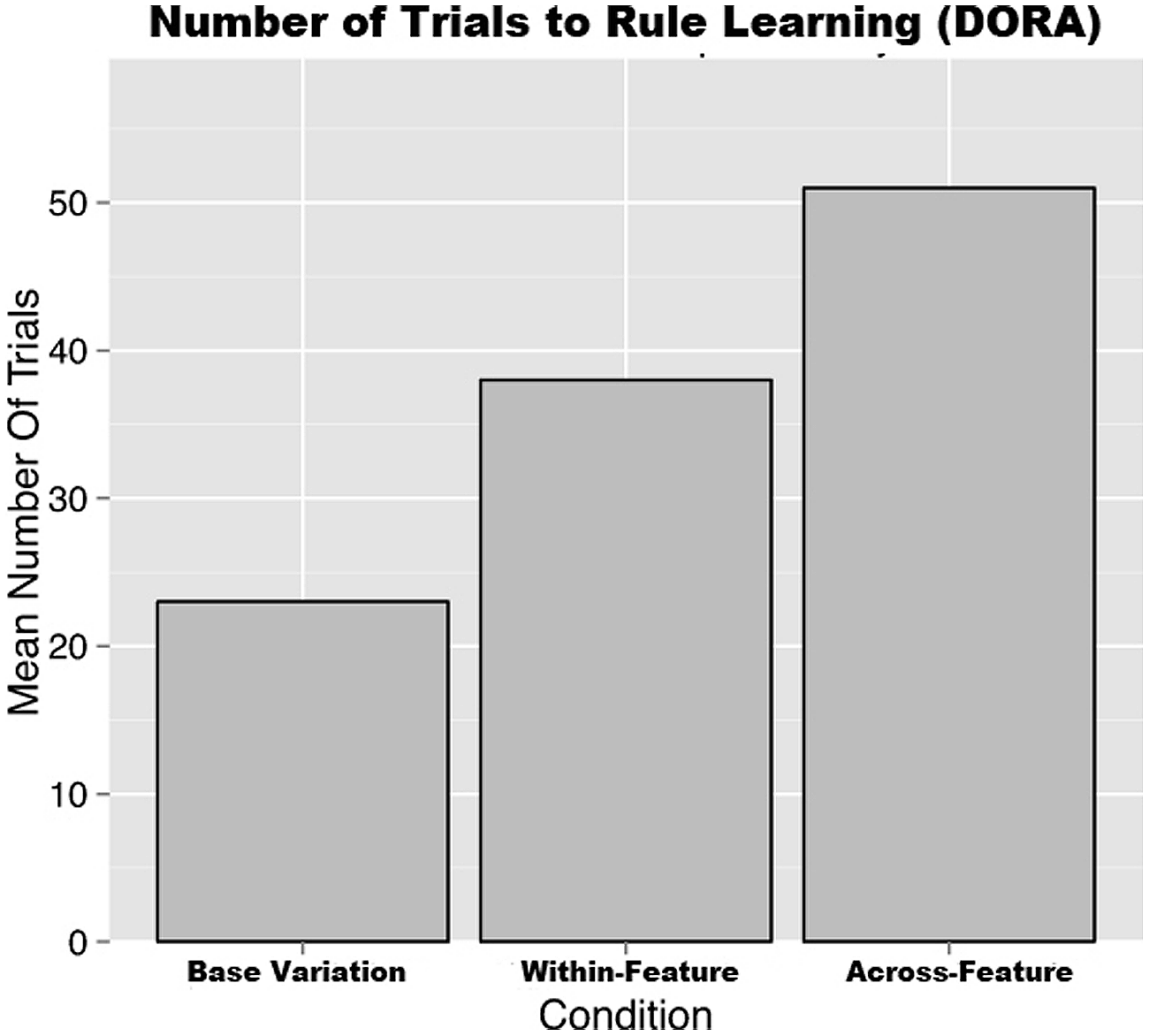
**DORA’s average number of trials taken to learn a rule**.

**Figure 8** depicts DORAs performance in comparison to the human data. Likewise, **Figure 11** depicts DORAs average number of trials per condition. A visual analysis of these graphs shows that DORA performed analogously to the human participants. In brief, these simulates support our hypothesis that increased variation in relational category-learning is more adversely affected when the variation comes from across- feature than when it comes from within a feature of interest.

## DISCUSSION

The goal of this paper has been to investigate the effects of feature variation in relational category-learning. We specifically reasoned that relational category-learning should be affected by the type of feature variation present in the training set, and we expected that across-feature variation would make learning more challenging than within-feature variation. This expectation was grounded in the fact that both relational learning and learning a category from feedback involve detecting similarities and differences across exemplars, and comparison across the training set (Doumas et al., 2008; Hammer et al., 2008, 2009a,b). Thus, the additional varying feature inherent to the across-feature variation condition offers an entirely new feature to rule out with regard to its potential diagnosticity for category membership, while within-feature variation does not. For example, in this experiment, if one were to rule out “shape” as the predicting factor of category membership, then the within-feature variation group would have no more features to have to consider and rule out. The across-feature variation group, by contrast, might still need to consider and rule out “color” as well, forcing them to take longer to hone in on the important *relational* structure. Markman and Maddox (2003) suggest a similar possibility in the case of feature-based category-learning tasks.

Given that it is often difficult to derive what mechanisms were involved in a given behavior, we used DORA as a way of testing whether such a process could produce our expected results and mirror the performance seen in our human participants. In short, it did.

To begin with, both our human and model data suggest that less variation is better overall. This conclusion is reflected in the learning rates, and in the fact that the base-variation condition had a much higher proportion of rule learners than did the other conditions. However, the results also suggest that within-feature and across-feature variation do not affect rule learning in the same way. To the point, the speed of rule acquisition among rule learners in the within-feature variation group was on par with that of the base-variation group, whereas the across-feature variation group showed much slower rule acquisition rates. This trend was also seen in the human reaction times during rule-learning, where the base-variation group was only significantly faster than the across-feature variation group. Interestingly though, once participants learned the rules, there was no difference among groups in their ability to generalize to novel exemplars. Thus, feature variation seems to affect rule learning more than rule application.

Thus, it is interesting that this study points out a similarity between feature-based and relational categories. A large body of literature has described relational categories as qualitatively and functionally unique from feature-based categories (e.g., Gentner, 2005; Gentner and Kurtz, 2005); however, the results of the present experiment suggest that both types of categorization are sensitive to feature variation, and both are sensitive to the type of feature variation exhibited in the training exemplars.

We do not suggest that this research is exhaustive by any means, and we acknowledge that open questions remain. For instance, the task used involved a rather low amount of variability in simple stimuli; it seems prudent to test whether the results found here will scale up to stimuli that are more complex or to greater amounts of variability. Furthermore, category-learning represents only one type of relational processing. It would be interesting to ask how different types of feature variability affect other tasks. For example, as discussed earlier, cross-mapping analogy problems are difficult because they use identical or highly similar objects in different roles across the base and the target analog. It seems reasonable to question how varying levels and types of feature variation between the base and target would affect mapping.

Despite open questions though, this paper has begun to explore the relationship between relational category-learning and different types of feature variation. We conclude that both quantity and quality of the variation in a training set can significantly affect learning, and that a reasonable explanation for this difference is a disparity in the representational cost associated with each type of variation. Thus, future relational category-learning paradigms may need to consider how and where they implement feature variation.

## Conflict of Interest Statement

The authors declare that the research was conducted in the absence of any commercial or financial relationships that could be construed as a potential conflict of interest.

## Footnotes

1 Featural distractors can make relational reasoning more difficult, at least in part, because of human WM constraints (Richland et al., 2006; Morrison et al., 2010). WM has often been proposed as an important component to relational reasoning (e.g., Halford et al., 1998). Specifically, mapping between two relations requires holding the relations in WM, and then finding structural alignments between the objects in them (Gentner, 1983). At the same time, mappings between similar features (and identical objects) must be actively inhibited, which taxes WM (Morrison et al., 2010). As a result, tasks that involve relational representations typically suffer when greater numbers of features are present (i.e., when the number of features that must be overlooked is increased; Doumas et al., 2008; Morrison et al., 2010).

2 While we label semantic units (e.g., “low,” “object”) for the purposes of explication, semantic units in DORA do not have any real ‘meaning’ *per se*, and operate like sub-symbolic units in traditional distributed representations (e.g., McClelland, 2009). All that matters for the purposes of DORA’s learning algorithm is that the system is capable of encoding something invariant across instances of the same relational concept (e.g., instances of *wider* or *above*; see Doumas et al., 2008).

3 Importantly, DORA learned all of the explicit predicate representations of different shapes [e.g., *circle*(*x*)], colors [e.g., *red*(*x*)], and spatial relations [e.g., *above*(*x*,*y*)] from examples of objects of various shapes and colors in various spatial configurations used in these simulations. The learning simulations DORA’s predicate learning algorithm (see Doumas et al., 2008), and proceeded just like various other predicate learning simulations we have run in several other studies (e.g., Doumas et al., 2008; Doumas and Hummel, 2010). We do not describe these simulations in detail here because DORA’s ability to learn new predicates is actually quite tangential to the main purpose of exploring how already learned properties can affect relational category-learning. We note them only because it is important to note that the representations used in the next part of the simulation were learnable by the model, and emerged as the natural consequence of model’s proposed predicate discovery algorithm. Excruciating details of how DORA learns new predicates can be found in Doumas et al., 2008).
